# Artificial Intelligence for the Diagnosis of Respiratory Diseases in Dogs and Cats: A Systematic Review

**DOI:** 10.3390/vetsci13020163

**Published:** 2026-02-07

**Authors:** Franklin Parrales-Bravo, Janio Jadán-Guerrero, Katherine Medina-Castro, Rosangela Caicedo-Quiroz

**Affiliations:** 1 Artificial Intelligence Research Group, Universidad Bolivariana del Ecuador, Km 5 ½ vía Durán—Yaguachi, Durán 092405, Ecuador; rcaicedoq@ube.edu.ec; 2 Grupo de Investigación en Inteligencia Artificial, Facultad de Ciencias Matemáticas y Físicas, Universidad de Guayaquil, Guayaquil 090514, Ecuador; katherine.medinac@ug.edu.ec; 3 Faculty of Computer Science, Complutense University of Madrid, Av. Séneca, 2, 28040 Madrid, Spain; 4 Centro de Investigación de Ciencias Humanas y de la Educación (CICHE), Universidad Tecnológica Indoamérica, Quito 170103, Ecuador

**Keywords:** assistance tools, pathology, pets, chest X-rays, internal medicine

## Abstract

Respiratory diseases represent a leading cause of veterinary consultations in dogs and cats, yet their detection remains challenging due to clinical variability and subjective interpretation of traditional diagnostic methods. In recent years, artificial intelligence (AI) has emerged as a promising tool to augment veterinary diagnostics through automated analysis of imaging and physiological data. This systematic review synthesizes and critically evaluates 24 studies published from 2019 onward that explore AI applications to support the detection of respiratory diseases in dogs and cats, focusing on three complementary modalities: audio-based (e.g., respiratory sounds), image-based (e.g., chest radiographs), and multimodal approaches. Our findings indicate that deep learning models, particularly convolutional neural networks (CNNs) and transformer architectures, achieve clinically relevant accuracy in detecting conditions such as cardiomegaly, alveolar patterns, and Brachycephalic Obstructive Airway Syndrome (BOAS). However, significant barriers remain, including data scarcity, lack of standardized datasets, and limited real-world validation. This review highlights the transformative potential of AI in veterinary respiratory diagnostics while underscoring the need for collaborative efforts in data sharing, methodological standardization, and clinical integration to realize its full impact in practice.

## Simple Summary

Diagnosing breathing problems in dogs and cats is often difficult because traditional methods rely heavily on a veterinarian’s personal judgment and experience. This review examines how artificial intelligence—computer systems that can learn from data—can help to support the detection of these illnesses more reliably. We analyzed 24 recent studies where artificial intelligence (AI) was used in three ways: listening to breathing sounds, reading chest X-rays and scans, and combining different kinds of data like those from sound and movement sensors. The results show that AI can spot serious conditions like heart enlargement and lung diseases with high accuracy. However, wider use is limited by a lack of shared animal health data and real-world testing in clinics. Overall, AI offers great promise to support veterinarians in making quicker, more consistent diagnoses, leading to better care and healthier lives for pets.

## 1. Introduction

Respiratory diseases are one of the leading causes of veterinarian visits in pets, especially dogs and cats [1], with conditions such as bronchitis, pneumonia, feline asthma, and brachycephalic obstructive airway syndrome (BOAS) representing a significant clinical burden in companion animal practice [2]. Difficulties in interpretation arise from several factors, including the subtle and often overlapping nature of respiratory sounds—such as distinguishing between wheezes, crackles, and stertor—which can indicate different underlying conditions like feline asthma, pneumonia, or Brachycephalic Obstructive Airway Syndrome (BOAS) [3]. Additionally, many respiratory conditions exhibit clinical signs similar to those of diseases affecting other organ systems, particularly cardiovascular disorders; for example, cardiogenic pulmonary edema can mimic radiographic and auscultatory findings of pneumonia, complicating accurate and timely diagnosis without advanced imaging or multimodal assessment [4,5]. These diseases present a significant challenge for diagnosis due to clinical variability and the reliance on professional experience in the interpretation of traditional tests [6].

Detection of these pathologies in a timely manner is essential to prevent disease progression, reduce complications, and improve animal welfare [1]; however, techniques such as clinical auscultation and radiographic interpretation can exhibit high variability between observers, particularly when abnormalities are subtle or in their initial stages [6,7]. For example, studies such as those by Banzato et al. [8] and Dumortier et al. [9] highlight how subjective interpretation of thoracic radiographs—even among experienced clinicians—can lead to inconsistent diagnoses of conditions like alveolar patterns or pulmonary abnormalities. Similarly, Oren et al. [10] note that traditional auscultation-based assessment of respiratory sounds in brachycephalic dogs is heavily dependent on examiner expertise, often resulting in diagnostic inconsistency. This inter-observer variability underscores the critical need for more objective, AI-supported tools to support detection in veterinary respiratory medicine. Given these constraints, there is a pressing need to systematically evaluate the current state of artificial intelligence (AI) applications [11] specifically tailored to veterinary respiratory diagnostics. Although several reviews have explored AI in veterinary medicine in general, few have focused on the early detection of respiratory diseases in dogs and cats—a critical gap given the clinical prevalence and diagnostic complexity of these conditions [1,12]. A focused review can help identify modality-specific advances, benchmark performance, and highlight translational challenges unique to companion animals.

AI has played a significant role as a tool to support medical diagnosis in pets through automated analysis of images and physiological signals [13,14,15]. In human medicine, numerous studies have demonstrated that machine learning and deep learning techniques can achieve performance comparable to or better than that of specialists in the detection of respiratory diseases [16,17,18]. In contrast, in the field of veterinary medicine, the application of these AI technologies is still limited due to the scarcity of large, well-annotated datasets [19,20], the anatomical and pathological diversity between multiple species [14,21], and the significant economic and infrastructural constraints of most veterinary practices [19]. In fact, unlike human healthcare, where data collection is often standardized and supported by major institutions, veterinary data is frequently fragmented between clinics with varying equipment and record-keeping practices [22]. This creates a substantial bottleneck for training robust generalizable models [23]. However, pioneering research is beginning to emerge that focuses on conditions such as canine pulmonary fibrosis or feline asthma, offering a promising glimpse into a future where AI-powered tools could become vital assistants in the veterinary clinic, helping to make earlier and more accurate diagnoses for our animal companions [14,24].

The purpose of this systematic review is to synthesize and critically evaluate the existing literature on artificial intelligence (AI) applications to support the detection of respiratory diseases in dogs and cats, with a specific focus on three diagnostic modalities:

- Audio-based approaches (e.g., respiratory sounds and vocalizations);
- Image-based methods (e.g., chest radiographs, CT scans);
- Multimodal integrations (e.g., combining audio, video, and sensor data).

Moreover, given the rapid evolution of deep learning architectures and the marked increase in veterinary AI publications due to COVID-19 pandemic [25], this review focuses on studies published from 2019 onward. This review aims to identify the types of clinical data used, the AI techniques most frequently employed, and the reported diagnostic performance, thereby highlighting the main opportunities and challenges for future research and clinical implementation in veterinary medicine.

This systematic review addresses the following research questions (RQs) for studies grouped by each analytical approach (audio-based, image-based, and multimodal AI):

- **RQ1: What are the techniques used?** What artificial intelligence (AI) and machine learning (ML) techniques—such as convolutional neural networks (CNNs), recurrent neural networks (RNNs), transformers, ensemble methods, or signal processing methods—are employed in audio-based, image-based, and multimodal diagnostic approaches for the detection of respiratory diseases in dogs and cats?
- **RQ2: What are the key findings?** What are the primary diagnostic performance outcomes (e.g., accuracy, sensitivity, specificity, AUC-ROC) reported in studies using each approach? What respiratory conditions (e.g., BOAS, cardiomegaly, alveolar patterns) are most commonly detected, and how reliable are these AI models in veterinary settings?
- **RQ3: What are the veterinary clinical implications?** How can AI-driven tools enhance veterinary practice in terms of diagnostic accuracy, workflow efficiency, detection, remote monitoring, and clinical decision support? What are the potential impacts on animal welfare, treatment outcomes, and veterinary resource allocation?

These questions systematically guide the evaluation of AI applications in veterinary respiratory diagnostics, ensuring a structured analysis of techniques, outcomes, implications, alignment with existing literature, and critical assessment of each diagnostic modality.

The remaining sections of the paper are structured as follows: Section 2 provides a review of related literature, positioning our work within the broader context of AI applications in veterinary medicine. Section 3 details the systematic methodology employed for study selection and data synthesis. Section 4 presents the findings categorized by diagnostic modality—audio-based, image-based, and multimodal AI approaches. Section 5 discusses the implications, challenges, and future directions of AI in veterinary respiratory diagnostics. Section 6 outlines the practical implications for both research and clinical practice. Section 7 acknowledges the limitations of this review and suggests future perspectives. Lastly, Section 8 encompasses concluding remarks, summarizing the potential and current constraints of AI in supporting the detection of respiratory diseases in dogs and cats.

## 2. Related Work

This section provides a comprehensive overview of existing literature on artificial intelligence in veterinary sciences, with a particular focus on diagnostic applications.

In Table 1, we present a summary of relevant review works on the topic of AI in veterinary sciences conducted in the last 5 years, indicating for each the main goal, findings, and differences in their approach compared to our study, which is narrowly focused on the application of AI for the detection of respiratory diseases specifically in dogs and cats, primarily using chest radiographs and respiratory sounds as data sources.

The studies summarized in Table 1 collectively aim to map, evaluate, and advance the integration of AI across the diverse landscape of veterinary medicine. Their goals range from providing comprehensive overviews of AI’s potential applications—spanning clinical practice, biomedical research, public health, and administration [19,25,31]—to offering practical educational guides for veterinary practitioners [28]. Several reviews focus specifically on diagnostic imaging, analyzing AI’s role in enhancing detection, classification, and segmentation across various modalities like radiology and ultrasound [13,27,30], while others adopt a broader, holistic perspective to explore AI’s transformative prospects in diagnostics, predictive medicine, personalized treatment, and drug development [14,19,31]. A subset compares AI applications between human and veterinary medicine [26] or assesses the feasibility and ethical considerations of AI deployment in clinical settings [27,30]. Despite their varied scopes, a common thread is the intention to synthesize existing knowledge, identify current trends and challenges—such as data scarcity, methodological heterogeneity, and the need for human oversight—and ultimately chart a path toward more effective, efficient, and ethically sound AI tools that support, rather than replace, veterinary professionals.

## 3. Methodology

This literature review was conducted using a systematic approach to identify peer-reviewed journal articles published between 2019 and 2025 that focus on the application of artificial intelligence (AI) for the detection of respiratory diseases in pets, particularly dogs and cats. The decision to restrict the review to studies published from 2019 onward is grounded in the rapid evolution of computer-aided veterinary diagnostics during this period due to COVID-19 [25]. In fact, the COVID-19 pandemic spurred increased research interest in AI-driven respiratory health monitoring, further expanding the volume and quality of relevant publications from 2019 onward. By focusing on this timeframe, this review ensures that the included works reflect contemporary applications, and clinical relevance essential for current and future AI applications in veterinary practice.

It is important to mention that the final report followed the PRISMA-ScR (Preferred Reporting Items for Systematic reviews and Meta-Analyses extension for Scoping Reviews) guidelines [32]. The protocol was pre-registered in the Open Science Framework (OSF) portal (Available in https://osf.io/tqbwd/overview?view_only=eb8578d80ef745cbbeb9d370ee8bf800) (accessed on 23 December 2025).

### 3.1. Databases and Search Strategy

Multiple electronic databases were searched, including PubMed, Scopus, IEEE Xplore, and Web of Science. They were selected for their coverage in the fields of veterinary medicine, biomedical engineering, and artificial intelligence applied to health. In addition, we have considered Google Scholar because it provides a comprehensive and broad-reaching search of scholarly literature across multiple disciplines and sources, including peer-reviewed papers, theses, books, and conference proceedings, which helps to capture a wider range of potentially relevant studies that may not be indexed in more specialized databases.

Table 2 presents the company names and addresses (city, country) of the databases and software used. The search strategy was designed to systematically identify studies applying artificial intelligence to support the detection of respiratory diseases in dogs and cats. Reflecting the three core diagnostic modalities under review—audio-based, image-based, and multimodal AI—the strategy incorporated targeted keywords and Boolean operators for each approach. For audio-based diagnostics, search terms included “respiratory sounds,” “cough detection,” and “lung sounds.” For image-based diagnostics, terms such as “thoracic radiograph,” “lung ultrasound,” and “CT scan” were combined with AI-specific terms like “CNN,” “ResNet,” and “U-Net.” For multimodal approaches, keywords included “multimodal AI,” “combined model,” and “fusion model” to capture integrative studies. The searches were adapted to the specific syntax of each database and were limited to articles published between 2019 and 2025, a period that coincides with the consolidation of deep learning in diagnostic veterinary applications. Table 3 presents the number of articles retrieved using each search strategy applied.

### 3.2. Inclusion Criteria

For the present study, we selected articles that met the following criteria:

- Studies published between 2019 and 2025.
- Original scientific articles published in indexed journals or peer-reviewed conference proceedings.
- Studies focused on dogs or cats.
- Research applying artificial intelligence techniques to analyze thoracic radiographs, respiratory sounds, or clinical data related to the respiratory system.
- Studies reporting diagnostic performance metrics, such as accuracy, sensitivity, specificity, or AUC-ROC.
- Publications available in English or Spanish.

### 3.3. Exclusion Criteria

In this study, we discarded the articles that contained any of the following points:

- Studies focused exclusively on human medicine without application or extrapolation to the veterinary field.
- Studies addressing non-respiratory pathologies.
- Studies using computational techniques without applying artificial intelligence or machine learning.
- Opinion pieces, editorials, abstracts without full text, or duplicate documents.
- Research without a clear description of the methodology or without a report of diagnostic results.
- Articles not written in English or Spanish.

### 3.4. Data Extraction and Synthesis

**Figure 1:**
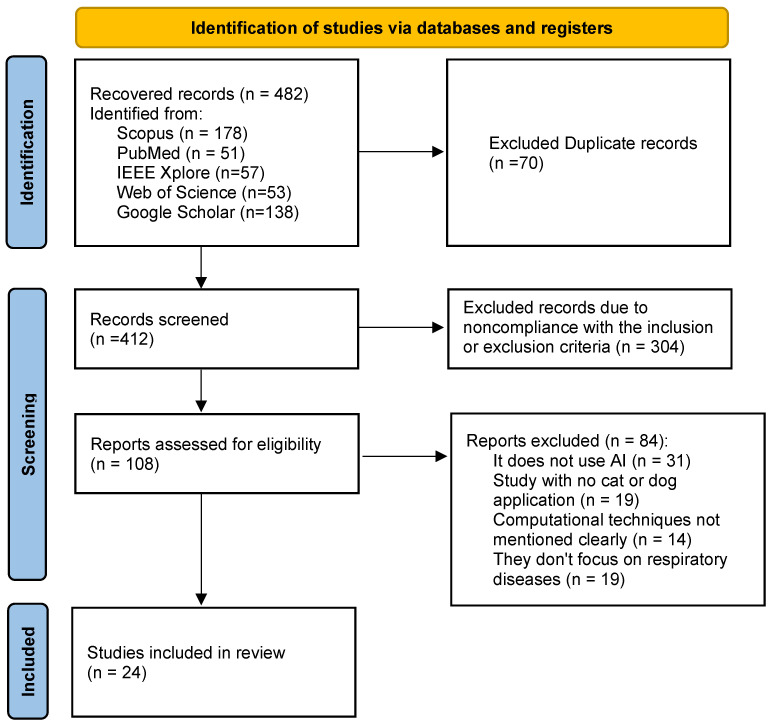
Flowchart of study selection according to the PRISMA guidelines.

Figure 1 presents the flowchart of study selection according to the PRISMA guidelines [32]. The selection process was carried out in several stages. Initially, the records were identified by searching the selected databases. Subsequently, duplicates were removed and a preliminary screening was performed based on the title and abstract. The potentially relevant studies were then assessed by reading the full text to determine their final eligibility.

## 4. Results

The literature search yielded a total of 558 potential articles; following a detailed screening process (as shown in Figure 1), 24 studies met the inclusion criteria, with 5, 13 and 6 related to audio-based, image-based and multimodal-based diagnostics, respectively. The following sections summarize the key findings, grouping the works by each diagnostic approach employed.

### 4.1. Audio-Based Diagnostic

This section focuses exclusively on research that uses respiratory sounds or vocalizations as primary data for the detection and assessment of conditions such as Brachycephalic Obstructive Airway Syndrome (BOAS) in dogs. Table 4 (and its continuation) summarizes the selected studies in the domain of audio-based AI diagnostics for respiratory diseases in commonly companion animals, specifically, Cats and Dogs. The included studies employ a range of AI techniques—from convolutional and recurrent neural networks to ensemble classifiers and signal processing methods—to analyze audio recordings captured via electronic stethoscopes or other acoustic sensors. Each entry outlines the study’s goal, sample characteristics, data type, AI methodology, key performance metrics, and veterinary implications, thus providing a structured overview of how audio-driven AI is currently being applied and validated in veterinary respiratory diagnostics.

Based on the studies summarized in Table 4, the application of AI for audio-based diagnosis of respiratory diseases in veterinary medicine has shown promising results, particularly in the detection and assessment of conditions such as Brachycephalic Obstructive Airway Syndrome (BOAS) in dogs. These studies utilize various AI techniques, including convolutional neural networks (CNNs), recurrent neural networks (RNNs), ensemble models, and signal processing methods, to analyze respiratory sounds and vocalizations. For instance, Karaslan et al. [33] developed a fully automatic voice analysis system capable of classifying dog vocalizations with up to 90% accuracy, using CNNs with features such as MFCC and STFT. This system enables automated behavioral and health monitoring, supporting stress and emotion assessment in clinical settings. Similarly, McDonald et al. [34] employed an RNN with GRU and attention mechanisms to detect stertor in brachycephalic dogs, achieving an AUC of 0.85, which facilitates accessible and objective BOAS screening through electronic stethoscopes and potential smartphone applications.

### 4.2. Image-Based Diagnostic

This section focuses on studies that employ thoracic imaging data—primarily radiographs, but also CT scans and ultrasound—as the main input for AI-driven diagnosis of respiratory conditions in dogs and cats. The works summarized in Table 5 utilize deep learning architectures such as convolutional neural networks (CNNs), ResNet, DenseNet, U-Net, and transformer-based models to perform tasks including classification, segmentation, and quality assessment of thoracic images. These approaches aim to support veterinarians by automating the detection of patterns such as alveolar infiltrates, pleural effusion, cardiomegaly, and pulmonary masses, as well as by providing objective measurements like Vertebral Heart Size (VHS) and Cardiothoracic Ratio (CTR). The table outlines each study’s objective, sample characteristics, imaging modality, AI technique, key performance outcomes, and potential clinical implications, offering a consolidated view of how image-based AI is currently advancing respiratory diagnostics in veterinary practice.

The studies compiled in Table 5 demonstrate that AI, particularly deep learning models, has achieved considerable success in analyzing thoracic images for respiratory disease detection in dogs and cats. Architectures such as ResNet-50, DenseNet-121, U-Net, and vision transformers have been widely employed for tasks ranging from multi-label classification of radiographic patterns to automated segmentation of lung fields and masses. Performance metrics reported are often clinically relevant, with many studies achieving AUC values above 0.8 or 0.9 for conditions like alveolar patterns, pleural effusion, pneumothorax, and cardiomegaly [8,38,43]. For example, Banzato et al. [8] reported AUCs greater than 0.9 for detecting alveolar patterns and pleural effusion in feline radiographs, while Burti et al. [43] achieved an AUC of 0.973 for detecting cardiomegaly in dogs using ResNet-101. Segmentation tasks have also shown high precision, with Jurgas et al. [44] reporting a Dice Similarity Coefficient of 0.91 for pulmonary mass segmentation in canine CT scans. Additionally, some studies have extended beyond detection to include image quality assessment, such as evaluating collimation, positioning, and exposure [39], which can help reduce non-diagnostic studies and improve workflow efficiency.

### 4.3. Multimodal-Based Diagnostic

This section examines studies that integrate multiple types of data—such as audio, video, sensor signals, and imaging—to enhance the detection and monitoring of respiratory conditions in dogs and cats. Multimodal AI approaches aim to overcome the limitations of single-modality systems by combining complementary information, thereby improving diagnostic robustness, accuracy, and clinical applicability. The works summarized in Table 6 employ a variety of AI techniques, including artificial neural networks (ANNs), convolutional neural networks (CNNs), and custom algorithms, to analyze integrative data from wearable devices, smartphones, and pressure sensors. Each entry outlines the study’s objective, sample characteristics, data types, AI methodology, key contributions, and veterinary implications, providing a structured overview of how multimodal AI is being developed and validated for respiratory health assessment in companion animals.

The integration of multimodal data through artificial intelligence represents a significant advancement in veterinary diagnostics, particularly for the detection and continuous monitoring of respiratory diseases in companion animals. Studies such as those by Withington et al. [48] and Angelucci et al. [50] illustrate how combining diverse data streams—including audio, video, motion, and sensor signals—can yield a more holistic and accurate assessment of an animal’s respiratory health. For instance, Angelucci et al. [50] demonstrated that video-based respiratory rate monitoring in sleeping dogs achieved high accuracy (RMSE = 1.1, MAE = 0.7), offering a low-cost, non-invasive method suitable for at-home monitoring and telemedicine applications. Similarly, Jarkoff [49] reported that a smart collar equipped with motion sensors and AI algorithms could estimate resting heart and breathing rates with minimal error (SMAPE 0.38% for heart rate, 1.42% for breathing rate), enabling continuous, real-time vital sign tracking outside clinical settings.

## 5. Discussion

Our systematic review successfully synthesized the emerging evidence on AI applications to support the detection of respiratory diseases in dogs and cats. The most important finding of this study is that, while promising, veterinary AI lags significantly behind its human medicine counterpart. Unlike human healthcare, where AI tools for respiratory diagnosis are more advanced and widely validated, veterinary applications are constrained by critical barriers such as data scarcity, lack of standardization, and limited real-world clinical integration. This discussion synthesizes the key opportunities and challenges across the three diagnostic modalities, highlighting areas requiring focused research, standardization, and validation.

### 5.1. Audio-Based Diagnostics: Beyond BOAS and Towards Generalized Respiratory Sound Analysis

Current audio-based AI research has effectively demonstrated its utility in objective screening for Brachycephalic Obstructive Airway Syndrome (BOAS), achieving notable accuracy through models analyzing laryngeal sounds and post-exercise respiratory patterns. Traditional auscultation and clinical evaluation can be subjective and variably performed, whereas AI models offer standardized, data-driven insights. In fact, respiratory sounds in dogs and cats can be challenging to differentiate due to overlapping acoustic characteristics between normal and abnormal sounds, as well as between different pathologies. Below, we clarify the physiological and anatomical origins of sounds that may lead to confusion in AI-based evaluation:

- Normal respiratory sounds (e.g., tracheal, bronchial, and vesicular sounds) arise from laminar airflow through open airways and are typically soft, low-pitched, and regular. Abnormal sounds such as stertor (originating from the nasopharynx) and stridor (originating from the larynx or trachea) are often higher-pitched and may be confused with normal turbulent airflow in brachycephalic breeds, where anatomical narrowing is common even in healthy individuals [54].
- Crackles (associated with pulmonary edema, fibrosis, or pneumonia) and wheezes (associated with airway obstruction, e.g., feline asthma) can be difficult to distinguish from artifacts (e.g., movement, panting, or environmental noise) or from normal inspiratory/expiratory sounds in anxious or panting animals [55].
- Breed-specific anatomical variations (e.g., elongated soft palate in brachycephalic dogs) can produce sounds that resemble pathological stertor, leading to false positives if models are trained on limited or non-representative datasets.

These overlapping acoustic profiles highlight the need for AI models to be trained on well-annotated, diverse datasets that include clear labels for sound origin (anatomical site) and context (rest, exercise, stress). Future work should also incorporate multimodal data (e.g., simultaneous video or spirometry) to disambiguate ambiguous acoustic patterns.

Futhermore, it is insightful to compare these AI-based audio diagnostic approaches with traditional, non-AI methods for BOAS assessment, such as structured owner questionnaires and standardized exercise tolerance tests (ETTs). For example:

- The study by Anyamaneecharoen et al. [56] utilized a detailed owner questionnaire and a 6-min walk test (6-MWT) to assess Brachycephalic Obstructive Syndrome (BOAS) severity in French Bulldogs. Their results showed a clear gradient: the normal group walked significantly farther (521±35m) than the moderate (422±37m) and severe (392±50m) BOAS groups. Furthermore, they found a strong negative correlation (*r* = −0.757, *p*< 0.001) between the 6-MWT distance and the owner-reported breathing sound scores, indicating that poorer exercise capacity closely aligns with more severe respiratory noise.
- Similarly, Reyes-Sotelo et al. [57] employed a combination of a 6-min walk and a 1000-m walk test to evaluate dogs of different cephalic biotypes. Their findings confirmed that brachycephalic dogs, especially those with BOAS grades 2 and 3 (G2, G3), covered significantly less distance in the 1000-m test and exhibited more pronounced physiological alterations (e.g., sustained low SpO_2_, elevated heart and respiratory rates, poor thermoregulatory recovery) compared to dolichocephalic and mesocephalic dogs. They also identified specific morphometric risk factors, such as muzzle length < 38 mm and nasal fold thickness ≥ 20 mm, associated with severe BOAS.

These traditional studies underscore the clinical value of exercise tests but also highlight their inherent limitations and risks. Tests like the 6-MWT or 1000-m walk, while informative, impose physical stress that can be hazardous for severely affected brachycephalic dogs, potentially triggering dyspnea, cyanosis, hyperthermia, or collapse. This is precisely where AI-based audio diagnostics present a transformative opportunity.

The AI models reviewed, such as those deployed by McDonald et al. [34] (AUC = 0.85 for BOAS detection from laryngeal sounds) and Oren et al. [10] (85% accuracy), demonstrate that AI can extract diagnostically rich information from respiratory sounds recorded under controlled or minimally stressful conditions, potentially even at rest or during mild activity. For example, an AI model trained on both acoustic data (e.g., resting or post-mild-exercise respiratory sounds) and the corresponding outcomes of standardized 6-MWT or 1000-m walk tests could learn to predict a dog’s functional exercise capacity and BOAS severity grade. A dog presenting with specific acoustic signatures (e.g., certain stertor patterns, spectral characteristics identified by FFT) could be algorithmically assessed as “high risk” for failing a strenuous exercise test, thus contraindicating the physical test itself. This approach would shift the paradigm from provoking a physiological crisis to diagnosing by predicting it through safe, passive monitoring.

The future of BOAS diagnosis lies not in choosing between AI and traditional methods but in their intelligent integration to support the diagnosis:

1. **AI for Triage and Continuous Monitoring:** AI tools could be deployed in-clinic for rapid, objective screening during routine exams or via wearable/smartphone technologies for at-home monitoring. They can provide an initial, risk-strratified assessment without stress.
2. **Traditional Tests for Calibration and Validation:** Well-established questionnaires and controlled, gentle walk tests (like the initial phase of a 6-MWT in a climate-controlled environment) remain crucial for validating AI models, gathering owner-reported outcomes, and assessing cases where AI predictions are uncertain.
3. **Morphometric Data as Context:** As shown by Reyes-Sotelo et al. [57], morphometric data (muzzle length, neck circumference) are strong risk indicators. Future multimodal AI systems could integrate audio analysis with simple anatomical measurements from images or 3D scans for a holistic risk assessment.

In summary, while traditional questionnaire- and exercise test-based methods provide a valuable clinical benchmark and correlate well with disease severity, they carry non-negligible risk and are dependent on owner compliance and environmental control. AI-based audio diagnostics offer a complementary pathway that is objective, scalable, and minimally invasive. The most promising clinical application is to develop these AI tools as predictive filters, capable of identifying dogs for whom traditional exercise tests would be of high risk or low diagnostic yield. By doing so, veterinary practice can enhance patient safety, support the detection of BOAS, and guide breeding decisions with greater precision and ethical responsibility, moving proactively toward a model of predictive, preventive, and personalized care for brachycephalic breeds.

### 5.2. Image-Based Diagnostics: The Imperative for Explainability and Seamless Clinical Integration

Deep learning models for thoracic radiograph and CT scan analysis have demonstrated robust performance in detecting specific pathological patterns, such as alveolar infiltrates, cardiomegaly, and pulmonary masses [8,37,43,44]. However, transitioning these models from research environments to reliable clinical tools requires addressing two interconnected challenges that extend beyond mere algorithmic accuracy: enhanced explainability and practical workflow integration.

While techniques like Gradient-weighted Class Activation Mapping (Grad-CAM) provide valuable visual heatmaps by highlighting regions of interest in an image [9], their output often remains abstract for the practicing veterinarian. There is a pressing need for more intuitive, clinically-grounded explanations that align directly with the spatial and descriptive reasoning veterinarians employ during radiographic interpretation. Future AI systems should be designed to generate reports that explicitly reference specific, recognizable anatomical landmarks, which are fundamental to veterinary image assessment. For instance, an AI tool could indicate that a detected interstitial pattern is primarily localized around the perihilar region [58] or that cardiomegaly is suggested by a vertebral heart score (VHS) exceeding established thresholds based on the cranial border of the 4th thoracic vertebra and the caudal cardiac silhouette [59]. Other critical reference sites include the costophrenic angles for assessing pleural effusion [60], the bronchovascular pattern for identifying interstitial disease [61], the diaphragmatic border for evaluating intrathoracic masses or herniation [62], and the tracheal axis for detecting mediastinal shifts [63]. By correlating AI findings with these standard anatomical reference points and pairing them with standardized textual descriptors of radiographic signs (e.g., “moderate alveolar pattern in the left caudal lung lobe” or “increased bronchial wall thickness extending to the peripheral airways”), AI systems can bridge the interpretability gap, fostering trust and facilitating more efficient clinical decision-making.

Furthermore, a significant limitation in the current evidence base is that the majority of validation studies are conducted on retrospective, often highly curated, single-center datasets. The real-world diagnostic performance of these models can degrade substantially due to factors commonplace in general practice but underrepresented in training data. These include vast variations in image quality (e.g., exposure, motion blur), suboptimal patient positioning artifacts, the presence of concurrent and complex pathologies in a single study, and the diverse range of digital radiography systems and techniques used across clinics [39,64]. This underscores the critical and non-negotiable need for robust, prospective validation studies conducted in diverse, real-world clinical environments. Historically, many diagnostic aids and algorithms have shown promising accuracy in controlled experimental settings but have demonstrated poor sensitivity, specificity, or generalizability when deployed in routine practice, failing to maintain performance across different patient breeds, sizes, clinical states, and institutional protocols [14,64,65]. Therefore, rigorous multi-center prospective trials are essential. These trials must not only evaluate diagnostic accuracy metrics (like AUC, sensitivity, specificity) on held-out, multi-institutional data but must also assess clinical utility—measuring outcomes such as reduction in time-to-diagnosis, influence on treatment planning, improvement in diagnostic confidence among general practitioners, and, ultimately, impact on patient prognosis.

To this end, future development must prioritize the creation of “clinician-in-the-loop” AI systems. These are not meant to be autonomous, but are designed as intelligent assistants. Such systems should go beyond simple detection to quantify prediction uncertainty [66], automatically flag technically suboptimal images (e.g., poor collimation, rotation) in real-time before interpretation [67], and provide ranked differential diagnoses accompanied by confidence scores and relevant clinical context. Achieving this vision requires prospective testing of seamless integration with existing veterinary practice infrastructure, primarily Picture Archiving and Communication Systems (PACS), to evaluate the tool’s true impact on radiologic workflow efficiency, diagnostic error reduction, and overall clinician confidence in everyday settings.

### 5.3. Multimodal AI: Data Fusion Strategies and the Challenge of Clinical Actionability

The importance of multimodal AI lies in its ability to overcome the limitations inherent in single-modality approaches. By fusing complementary data sources—such as audio recordings for respiratory sounds, video for respiratory effort, and inertial sensors for movement and cardiac activity—AI systems can enhance diagnostic reliability, reduce false positives, and capture subtle physiological changes that may indicate any disease. Withington et al. [48] highlighted how pressure sensor data from medical detection dogs could be classified using artificial neural networks to automate behavioral responses, reducing cognitive load and training time. This approach not only supports diagnostic accuracy but also paves the way for automated, non-invasive monitoring systems that can operate in naturalistic environments, such as the home or shelter.

In practical veterinary use, multimodal AI tools offer several key benefits: they enable continuous monitoring of at-risk animals (e.g., brachycephalic breeds prone to BOAS), support telemedicine and remote consultations by providing objective data to veterinarians, and facilitate preventive care through longitudinal tracking of respiratory trends. For example, systems that combine audio and video inputs, as validated by Angelucci et al. [53], allow owners to participate actively in their pet’s health management using everyday devices like smartphones. Moreover, wearable technologies like the smart collar studied by Jarkoff [49] can alert caregivers to deviations from normal respiratory patterns, prompting timely veterinary intervention. These innovations are particularly valuable in settings with limited access to specialist care, as they provide scalable, user-friendly tools for respiratory assessment without requiring specialized equipment or frequent clinic visits.

Despite these promising developments, challenges remain in the widespread adoption of multimodal AI in veterinary practice. These include the need for standardized data collection protocols [14], integration into existing clinical workflows [68], and validation across diverse breeds and environments [69]. Future research should focus on creating shared multimodal datasets, improving model interpretability, and conducting multi-center clinical trials to ensure robustness and generalizability. Nevertheless, the current evidence underscores the transformative potential of multimodal AI to enhance respiratory disease detection, improve animal welfare, and support veterinarians with actionable, data-driven insights.

### 5.4. Bridging the Data Scarcity Gap: Leveraging Human Medicine and Advanced Learning Paradigms

A fundamental and persistent challenge across all AI modalities in veterinary diagnostics is the acute scarcity of large-scale, high-quality, and consistently annotated datasets [19,30]. Unlike human medicine, where initiatives like MIMIC-CXR [70] or the NIH Chest X-ray dataset [71] provide hundreds of thousands of labeled images, veterinary data remains fragmented, institution-specific, and often proprietary. This data paucity severely restricts the development, validation, and generalizability of deep learning models, which are inherently data-hungry. To overcome this critical bottleneck and unlock the full potential of veterinary AI, a strategic shift toward data-efficient methodologies is imperative. Future research must focus on two synergistic pathways: leveraging cross-domain knowledge and pioneering advanced, resource-conscious learning paradigms.

The first strategic pathway involves aggressive exploration of cross-species transfer learning and sophisticated domain adaptation techniques. In facr, the anatomical similarities in thoracic structures between humans and companion animals, especially in pathology manifestations like pleural effusion or pneumothorax [72], can provide a foundation for pre-training models on vast human data before fine-tuning on smaller veterinary sets. Pioneering studies, such as the work by Celniak et al. [47], have validated the feasibility of this approach. Their model, pre-trained on a heterogeneous mix of over 500,000 human and canine radiographs using self-supervised learning, demonstrated improved performance on veterinary-specific classification tasks after fine-tuning. This paradigm allows models to learn universal features of disease presentation—such as texture, shape, and spatial relationships—from the vast, richly annotated repositories of human medical imaging before specializing in the veterinary domain. Future work should extend this concept beyond radiography to other modalities, such as adapting models trained on human lung sound databases or human wearable sensor data for veterinary audio and physiological monitoring applications. The key will be developing robust domain adaptation algorithms that can effectively minimize the “domain shift” caused by anatomical differences (e.g., thoracic conformation in brachycephalic breeds) and imaging protocol variations.

The second, equally critical pathway is the dedicated adoption of next-generation machine learning frameworks designed for learning from limited supervision. Relying solely on supervised learning with expert-labeled data is unsustainable for the veterinary field. Instead, the community must invest in:

- **Self-Supervised Learning (SSL):** SSL algorithms can learn powerful representations by solving “pretext tasks” on unlabeled data, such as predicting the rotation of an image or reconstructing masked parts of a spectrogram. Veterinary clinics generate terabytes of unlabeled images and audio recordings daily. SSL can transform this untapped resource into a pre-training goldmine, creating foundation models that encode general veterinary-relevant features without a single diagnostic label [73].
- **Contrastive Learning:** This technique learns by contrasting similar (positive) and dissimilar (negative) data pairs. It is exceptionally effective for learning robust representations that are invariant to nuisance variations (e.g., different X-ray machine exposures, variable stethoscope placement) while being sensitive to pathological differences. This is crucial for building models that perform consistently across diverse clinical settings [74].
- **Few-Shot and Meta-Learning:** These paradigms aim to train models that can learn new diagnostic tasks from only a handful of examples. This is directly applicable to rare respiratory conditions in veterinary medicine or for quickly adapting a general model to a specific clinic’s patient demographics or imaging equipment [75,76].

Prioritizing these advanced paradigms will catalyze a shift from a dependency on massive, curated datasets toward intelligent, efficient learning from available data. The ultimate goal is to build AI systems that are not only accurate but also data-frugal, adaptable, and inherently robust to the heterogeneity of real-world veterinary practice. This strategic focus on bridging the data scarcity gap is not merely a technical improvement but a foundational requirement for the equitable, widespread, and clinically impactful deployment of AI in veterinary respiratory medicine.

### 5.5. Pathway to Clinical Deployment: Validation, Standardization, and Ethical Implementation

The transition of AI models from promising research to reliable, trusted tools in daily veterinary practice represents a significant translational challenge. This pathway requires a comprehensive, multi-faceted framework that extends far beyond demonstrating high accuracy on retrospective datasets [77]. The cornerstone of this framework is the shift from internal validation to rigorous external, multi-center prospective trials [19,30]. Such studies must be designed to evaluate models on heterogeneous, real-world data that reflects the full spectrum of clinical variability—including differences in imaging equipment, patient positioning, concurrent pathologies, and operator skill [39,64]. These trials should move beyond traditional performance metrics (e.g., AUC, sensitivity, specificity) to include robust assessments of clinical utility and impact. This involves measuring tangible outcomes such as reduction in diagnostic turnaround time, influence on clinical decision-making and treatment plans, improvement in diagnostic confidence among general practitioners, and, ultimately, positive effects on patient prognosis and welfare [14,31]. The history of diagnostic aids in both human and veterinary medicine underscores that high performance in controlled settings does not guarantee utility in the chaotic clinical environment; therefore, prospective validation is non-negotiable [65].

Concurrently, there is an urgent, community-wide need for standardization in both data acquisition and evaluation. The current heterogeneity in how data is collected (e.g., audio recording settings, radiographic views) and how results are reported (e.g., varying performance metrics, lack of clarity on test set composition) severely hampers meaningful comparison between studies and slows collective progress [27,30]. The development and adoption of veterinary-specific standards—such as consensus protocols for respiratory sound recording, guidelines for AI-optimized radiographic positioning, and standardized reporting checklists for AI studies (akin to human medicine’s CLAIM checklist)—are essential. Such standardization will ensure data quality, facilitate the creation of shared, high-quality datasets, and enable the aggregation of evidence across research groups [68].

Finally, and critically, the ethical dimensions of AI deployment must be proactively and transparently addressed to build trust and ensure responsible use. Key issues include:

- Algorithmic Bias and Fairness: Models must be evaluated for performance disparities across different dog and cat breeds, sizes, and ages to prevent inequitable care [28,31]. A model that performs well on Labrador retrievers but poorly on French Bulldogs would be clinically harmful and ethically untenable.
- Data Privacy and Security: Clear protocols must govern the collection, storage, and use of patient and client data, ensuring compliance with data protection regulations and maintaining client trust [19].
- Defining the AI’s Role: It must be unequivocally communicated that AI serves as a decision-support tool, augmenting rather than replacing veterinary clinical judgment and expertise. Guidelines are needed to define appropriate use cases, limitations, and the necessity of human oversight, particularly for high-stakes decisions [28,31,69].
- Transparency and Explainability: As discussed in Section 5.2, providing interpretable outputs that align with veterinary reasoning is not just a technical issue but an ethical imperative for informed use.

Successfully navigating the pathway to clinical deployment requires that technological advancement be matched by a commitment to rigorous validation, community standardization, and ethical stewardship. Establishing trust with veterinarians, technicians, and pet owners is as vital as algorithmic performance for the sustainable integration of AI into veterinary medicine.

## 6. Implications for Future Research and Clinical Veterinary Practice

Based on the systematic review presented in the present systematic review, the implications for future research and clinical veterinary practice are substantial and interconnected. They can be summarized in two key areas:

### 6.1. Implications for Future Research

Future research must pivot from proof-of-concept studies to translational work that bridges the gap to clinical utility. We consider the following key priorities:

- Data Infrastructure and Standardization: There is an urgent need for large-scale, collaborative efforts to create shared, well-annotated veterinary datasets. Research should focus on establishing standardized data acquisition protocols (e.g., for audio recording, radiographic positioning) to ensure data quality and model generalizability. Advanced learning paradigms like self-supervised and cross-species transfer learning should be aggressively explored to overcome data scarcity by leveraging unlabeled archives and human medical databases.
- Clinical and Technical Validation: Research must move beyond retrospective, single-center validation. Multi-center, prospective trials are essential to evaluate AI performance on heterogeneous, real-world data with variable quality and concurrent pathologies. Studies should assess not just diagnostic accuracy but also clinical utility metrics, such as impact on time-to-diagnosis, treatment decisions, and patient outcomes. For multimodal AI, research must develop and test intelligent data fusion strategies and clinical correlation engines to translate complex signals into actionable insights.
- Explainability and Human–AI Collaboration: Developing intuitive explainability tools that align with veterinary clinical reasoning is critical for trust and adoption. Future studies should design “clinician-in-the-loop” systems that quantify uncertainty, flag suboptimal data quality, and provide differential diagnoses. Research must also formally address ethical implementation, including studies on algorithmic bias across breeds and the development of guidelines for AI’s role as a decision-support tool.

### 6.2. Implications for Clinical Veterinary Practice

For practicing veterinarians, the evolution of AI promises to augment capabilities and reshape aspects of clinical workflow, but requires careful integration. We consider the following implications:

- Enhanced Diagnostic Support and Accessibility: AI tools will likely become vital decision-support aids, particularly in settings without specialist radiologists or during emergency hours. They can provide rapid, objective screening (e.g., for cardiogenic pulmonary edema), automate tedious measurements (e.g., Vertebral Heart Size), and assess radiographic quality in real-time to reduce repeats. This can improve diagnostic confidence, consistency, and detection for general practitioners.
- Shift Towards Proactive and Remote Monitoring: The rise of wearable sensors and multimodal AI enables a paradigm shift from reactive to proactive, continuous health monitoring. Veterinarians can leverage data from smart collars or owner-collected smartphone videos for longitudinal tracking of at-risk patients (e.g., brachycephalic breeds, cardiac cases). This supports earlier intervention, enhances telemedicine consultations with objective data, and empowers preventive care and owner engagement.
- Necessity for New Skills and Critical Engagement: Successful adoption requires veterinarians to develop digital literacy to critically evaluate AI outputs, understanding their limitations and potential biases. The profession must engage in shaping these tools, ensuring they address real clinical needs and integrate seamlessly into practice workflow. Ultimately, AI will not replace clinical expertise but will augment it, allowing veterinarians to focus more on complex decision-making, client communication, and hands-on care.

## 7. Limitations and Future Perspectives

This review has certain limitations that should be considered when interpreting its conclusions. First, the included studies are predominantly proof-of-concept in nature, often based on retrospective data from single institutions. This limits the generalizability of the findings to broader, more diverse clinical settings. Second, the heterogeneity in study designs, AI methodologies, and performance metrics across the reviewed literature makes direct comparisons and definitive conclusions about the superiority of any single approach challenging. Third, the scope of the review, focused on studies from 2019 onward, while capturing recent advancements, may not encompass all foundational work in the field.

The path forward requires addressing these limitations. Future research should prioritize the development of large, well-annotated, and shared veterinary datasets to improve model generalizability. There is a critical need for standardized protocols in data acquisition (e.g., for audio recordings and radiographic imaging) and performance reporting. Moreover, advancing beyond technical validation, rigorous multi-center prospective trials are essential to evaluate the real-world clinical utility, workflow integration, and impact on patient outcomes of these AI tools. Exploring advanced learning paradigms, such as self-supervised and cross-species transfer learning, could help overcome data scarcity. Finally, the ethical dimensions of deployment—including algorithmic bias, data privacy, and the clear definition of AI’s role as a decision-support aid—must be proactively addressed to ensure responsible and trusted adoption.

## 8. Conclusions

Based on this systematic review, the application of artificial intelligence (AI) to support the detection of respiratory diseases in dogs and cats shows significant promise, particularly through audio-based, image-based, and multimodal diagnostic approaches. The main findings indicate that deep learning models—such as convolutional neural networks (CNNs), ResNet, and U-Net—can achieve clinically relevant accuracy in specific tasks, such as analyzing chest radiographs for patterns like cardiomegaly and alveolar infiltrates and classifying respiratory sounds for conditions like Brachycephalic Obstructive Airway Syndrome (BOAS). These results suggest AI’s potential to serve as a supportive tool that can enhance diagnostic consistency, reduce observer variability, and aid in early intervention within veterinary practice.

In conclusion, AI represents a promising and transformative complementary tool for respiratory disease diagnosis in pets. However, its successful and reliable integration into clinical workflows is not yet realized. The transition from promising research to practical clinical application depends on a concerted effort to overcome the existing methodological, data-related, and validation barriers identified in this review.

## Figures and Tables

**Table 1:** Summary of recent literature reviews on AI in veterinary medicine, highlighting scope, focus, and differences with the present review on AI for respiratory disease detection in dogs and cats.

Authors	Year	Goal	Main Findings	Differences with Our Review
Gomes et al. [14]	2025	Review multimodal AI applications in veterinary diagnostics, identify trends, challenges, and future directions.	Multimodal AI integrating imaging, text, audio, and video improves diagnostic accuracy and workflow efficiency; common fusion strategies include early, late, and hybrid fusion; challenges include data scarcity, computational limits, adoption barriers, and ethical issues.	Broad focus on all veterinary diagnostic applications and multimodal data integration, while our review is systematically focused on the detection of respiratory diseases in dogs and cats across three complementary modalities: audio-based (e.g., respiratory sounds), image-based (e.g., chest radiographs), and multimodal AI approaches.
Rubini et al. [26]	2025	Compare AI in chest radiography between human and veterinary medicine.	AI improves diagnostic speed/accuracy; human oversight remains essential; veterinary AI is limited by small datasets, anatomical variability, and lack of research.	Focuses exclusively on chest radiography, while our review includes multiple data sources (e.g., respiratory sounds) and is specific to the detection of respiratory diseases in dogs and cats.
Bouchemia et al. [27]	2023	To review the literature and assess the feasibility, challenges, and applications of AI across all areas of veterinary medicine.	AI is applied in diagnostics (23.6%), education (11.4%), epidemiology (10.6%), animal production (11.2%), pathology (6.8%), animal health/welfare (7.7%), microbiology (4.1%) and other areas; diagnostic accuracy can reach 95% with faster results, but outcomes should support, not replace, veterinarians.	Broad scope covering all veterinary applications; not focused on detection, respiratory diseases, or specific data types (e.g., radiographs, respiratory sounds) like our review.
Appleby & Basran [28]	2022	Provide an introductory overview of AI concepts, methods, and considerations for veterinary practitioners, focusing on diagnostic imaging and general implementation challenges.	AI (especially ML and deep learning) can enhance diagnostic imaging through detection, segmentation, and classification tasks. Veterinarians play key roles in defining use cases, ensuring data quality, ownership, and ethical deployment. Highlights need for open data, regulatory frameworks, and client acceptance.	Our review is a systematic analysis focused exclusively on AI for the detection of respiratory diseases in dogs and cats using chest radiographs and respiratory sounds. The other paper is a general educational overview of AI in veterinary medicine, with broad coverage of concepts and imaging applications, not limited to a specific disease or data modality.
Akbarein et al. [25]	2025	Provide a comprehensive narrative review of AI applications across veterinary sciences, categorized into four domains: clinical practice, biomedical research, public health, and administration.	AI is widely used in veterinary medicine, with CNN, SVM, RF, ANN, DT, KNN, and Naive Bayes being the most common algorithms. Clinical practice (especially disease diagnosis and imaging) and administration are the most researched areas. AI improves diagnostics, resource management, and animal welfare.	Our review is systematically focused on the detection of respiratory diseases in dogs and cats, using primarily chest radiographs and respiratory sounds. The other review is broad, covering all veterinary AI applications across species and domains without disease-specific or modality-specific focus.
Basran & Appleby [29]	2022	Overview AI potential in veterinary medicine	AI can improve diagnostic accuracy, efficiency in imaging, behavior monitoring, and decision support; highlights radiomics, wearables, NLP, and CNNs	Focuses broadly on veterinary AI applications; our review is systematically focused on the use of AI for supporting the detection of respiratory diseases in dogs and cats, using primarily chest radiographs and respiratory sounds.
Burti et al. [30]	2024	To review the current state, applications, and ethical considerations of AI in veterinary diagnostic imaging across multiple modalities (radiology, ultrasound, CT, MRI).	AI is applied in various imaging modalities for detection, classification, and segmentation of diseases, achieving high diagnostic accuracy in specific tasks (e.g., ResNet-50 AUC >0.8 for thoracic findings). AI serves as a decision support tool, not a replacement for veterinarians. Ethical concerns include data bias, transparency, and the need for human oversight.	Focuses broadly on diagnostic imaging across all body systems and species; not specific to detection, respiratory diseases, or non-imaging data (e.g., respiratory sounds) like our review.
Sharun et al. [31]	2024	To explore the transformative prospects, applications, challenges, and ethical considerations of AI across the entire spectrum of veterinary science.	AI enhances diagnostics, predictive medicine, personalized treatment, remote monitoring, telemedicine, research, and drug development in veterinary care. Examples include high diagnostic accuracy in thoracic radiograph analysis (e.g., 92.3% for cardiogenic pulmonary edema) and the use of AI for outbreak prediction and antimicrobial resistance.	Broad, holistic review covering all veterinary domains (diagnostics, treatment, monitoring, drug discovery, etc.); not focused specifically on the detection of respiratory diseases in dogs and cats or on specific data modalities like chest radiographs and respiratory sounds.
Akinsulie et al. [19]	2024	Provide a perspective on the potential applications of AI in veterinary clinical practice and biomedical research, covering areas such as disease diagnosis, zoonotic monitoring, epidemiology, surgery, AMR, cancer, genomics, and drug development.	AI can enhance disease diagnosis, zoonotic surveillance, epidemiology, precision breeding, patient monitoring, surgery, and biomedical research (AMR, cancer, genomics, vaccine/drug design). Highlights use of radiomics, ML models, and AI tools (e.g., FluSPred, VIDHOP) in veterinary contexts.	Our review is a systematic analysis focused exclusively on AI for the detection of respiratory diseases in dogs and cats using chest radiographs and respiratory sounds. The other paper is a broad perspective covering all veterinary AI applications across many clinical and research domains without disease-specific or modality-specific focus.
Pereira et al. [13]	2023	Provide a practical overview of AI, ML, and DL in veterinary imaging for professionals, reviewing existing literature in small animal imaging.	Supervised learning and CNNs are most common; AI applied across body systems (musculoskeletal, thoracic, nervous, abdominal); commercial tools like PicoxIA and Vetology exist. Thoracic imaging is most studied.	Broad overview of AI in all veterinary imaging domains, not limited to respiratory diseases; does not focus on detection, multimodal integration, or systematic review methodology.

**Table 2:** Details of scientific databases considered in this review.

Database	Company/Organization	Address	Software
PubMed	NCBI/NLM (USA)	8600 Rockville Pike, Bethesda, MD, USA	https://pubmed.ncbi.nlm.nih.gov/advanced/ (accessed on 23 December 2025)
Scopus	Elsevier	Radarweg 29, Amsterdam, Netherlands (HQ); 230 Park Ave, New York, NY, USA	https://scopus.com/ (accessed on 23 December 2025)
Web of Science	Clarivate Analytics	1500 Spring Garden St, Philadelphia, PA, USA	https://www.webofscience.com/ (accessed on 23 December 2025)
IEEE Xplore	Institute of Electrical and Electronics Engineers (IEEE)	445 Hoes Lane, Piscataway, NJ, USA	https://ieeexplore.ieee.org/ (accessed on 23 December 2025)
Google Scholar	Google LLC	1600 Amphitheatre Parkway, Mountain View, CA, USA	https://scholar.google.com/ (accessed on 23 December 2025)

**Table 3:** Number of studies retrieved per diagnostic approach and database using the systematic search strategy.

Approach	Search Strategy	Database	Results
Acoustic-based	(“respiratory sounds” OR “cough detection” OR “lung sounds”) AND (“dogs” OR “cats” OR “canine” OR “feline”) AND (“AI” OR “machine learning” OR “deep learning”)	Scopus	35
		PubMed	12
		IEEE Xplore	18
		Web of Science	22
		Google Scholar	40
Image-based	(“thoracic radiograph” OR “lung ultrasound” OR “CT scan”) AND (“dogs” OR “cats” OR “canine” OR “feline”) AND (“AI” OR “CNN” OR “ResNet” OR “DenseNet” OR “U-Net”)	Scopus	42
		PubMed	25
		IEEE Xplore	10
		Web of Science	17
		Google Scholar	55
Multimodal-based	(“multimodal AI” OR “combined model” OR “fusion model”) AND (“respiratory”) AND (“dogs” OR “cats” OR “canine” OR “feline”)	Scopus	8
		PubMed	3
		IEEE Xplore	6
		Web of Science	2
		Google Scholar	15
		Total	482

**Table 4:** Studies related to AI to support audio-based diagnosis of respiratory diseases in dogs and cats.

Authors/Year	Goal	Sample	Data	Technique	Reference Exam	Contribution	Implications
Karaslan et al. (2024) [33]	Develop a fully automatic voice analysis system to detect and classify dog vocalizations (barking, howling)	103 audio samples (barking: 46, howling: 57)	Dog vocalizations (barking, howling) from “Audio Cats and Dogs” dataset	CNN (AlexNet, DenseNet, EfficientNet, ResNet50, ResNet152) with STFT, MFCC, LFCC features	Manual annotation of barking and howling in audio recordings (ground truth labels)	Best accuracy: 90% (DenseNet with Mel spectrograms, AlexNet with MFCC, EfficientNet with LFCC); automated segmentation and classification system	Enables automated behavioral and welfare monitoring in dogs; supports vocal pattern analysis for stress, emotion, and health assessment in veterinary settings.
McDonald et al. (2024) [34]	To automatically detect and grade stertor in brachycephalic dogs using laryngeal sound recordings for BOAS screening.	341 dogs (665 recordings)	Laryngeal electronic stethoscope recordings (4 kHz, 16-bit) with veterinarian-assessed stertor/BOAS labels	Recurrent Neural Network (RNN) with GRU and self-attention	The Respiratory Functional Grading (RFG) scheme performed by expert veterinarians	AUC = 0.85 for BOAS detection; sensitivity = 71%, specificity = 86%. Model can detect moderate/severe stertor from spectrograms despite noise.	Enables accessible, objective BOAS screening via electronic stethoscope; supports detection, treatment decisions, and breeding advice; potential for owner-led monitoring via smartphone apps.
Oren et al. (2023) [10]	To use machine learning for objective analysis of respiratory sounds in brachycephalic dogs for BOAS diagnosis	148 dogs (69 Pugs, 79 dogs of other brachycephalic breeds); 366 audio samples	Audio recordings via electronic stethoscope during standardized exercise test (rest and post-exercise)	Ensemble model: Majority Voting classifier combining KNN (local/global) and Decision Tree; openSMILE features	The outcome of a standardized BOAS fitness test (Pass/Fail) based on functional grading criteria (vital parameters, breathing noises, respiratory effort, cyanosis)	Achieved 85% accuracy in classifying BOAS test results using post-exercise sounds in Pugs; 83% accuracy in detecting laryngeal sounds (stridor)	Provides an objective, data-driven tool for BOAS assessment, reducing subjectivity and time of traditional auscultation; supports diagnosis and standardized evaluation across breeds
Dimopoulou et al. (2024) [35]	To investigate the use of respiratory signal analysis to assess severity of BOAS in dogs.	117 dogs (105 brachycephalic, 12 non-brachycephalic)	Laryngeal electronic stethoscope recordings (20–2000 Hz) pre- and post-exercise	Signal analysis (Fast Fourier Transform) and statistical analysis (ANOVA, ROC)	The validated Respiratory Functional Grading (RFG) scheme, assigning a BOAS severity grade (0–3) based on clinical examination	Seven sound variables significantly associated with BOAS grade. “Valley 1” best predictor post-ET (AUC = 87.8%); BOAS severity linked to greater magnitude in 0–1000 Hz and stronger low-frequency contribution (170–260 Hz).	Provides an objective, non-invasive tool for BOAS severity assessment; could aid in clinical decision-making, post-surgical monitoring, and breeding selection; potential for integration into clinical practice with technological development.
Lee et al. (2025) [36]	Develop a deep learning-based tool to evaluate the severity of mitral regurgitation (MR) in dogs with Myxomatous Mitral Valve Disease (MMVD) using digital stethoscope recordings.	460 dogs	Phonocardiogram signals (digital stethoscope)	CNN6, PaSST, ResNet38 with Fbank & mel spectrogram features	The Mitral INsufficiency Echocardiographic (MINE) score, a composite echocardiographic metric used to categorize MR severity	CNN6-Fbank achieved 94.12% accuracy, 97.30% specificity. Model outperformed PaSST & ResNet38 in classifying mild, moderate, severe MR.	Provides a non-invasive, rapid, cost-effective tool for the screening and monitoring of MR in dogs, aiding in timely clinical decision-making and reducing reliance on expensive echocardiography.

**Table 5:** Studies related to AI to support image-based diagnosis of respiratory diseases in dogs and cats.

Authors/Year	Goal	Sample	Data	Technique	Reference Exam	Contribution	Implications
Banzato et al. (2021a) [8]	To develop and test an AI-based CAD algorithm for automatic classification of common radiographic findings in feline thoracic radiographs.	1062 LL radiographs	Feline thoracic radiographs from two institutions, labeled for radiographic findings	Multi-label CNN (ResNet-50 and Inception V3)	Consensus interpretation by two experienced radiologists.	High accuracy (AUC > 0.9) for alveolar pattern, bronchial pattern, pleural effusion; moderate for cardiomegaly (AUC > 0.7); low for mass detection (AUC ∼ 0.5–0.6). No significant difference between architectures.	Potential to assist veterinarians in emergency settings by quickly identifying critical findings (e.g., pneumothorax, pleural effusion). Can be integrated into clinical workflow to reduce interpretation errors, especially in non-specialist settings.
Banzato et al. (2021b) [37]	To develop and test a multi-label deep CNN for automatic classification of common radiographic findings in canine thoracic radiographs and evaluate its generalization ability.	3063 LL (Data Set 1) + 776 LL (Data Set 2) total: 3839 LL radiographs	Canine thoracic radiographs from a single institution, two acquisition systems; labeled for multiple radiographic findings	Multi-label CNN (ResNet-50 and DenseNet-121)	Consensus interpretation by three experienced veterinary radiologists.	ResNet-50 achieved AUC > 0.8 for alveolar pattern, cardiomegaly, megaoesophagus, pleural effusion, pneumothorax, and unremarkable. Lower performance for bronchial/interstitial patterns and mass detection. ResNet-50 outperformed DenseNet-121 in generalization for certain findings.	Potential to assist veterinarians in daily practice by reducing interpretation errors, especially for non-specialists. Could improve workflow efficiency and diagnostic accuracy in clinics with limited access to specialist radiologists.
Saxena et al. (2023) [38]	To develop a novel method for classifying canine thoracic radiographs using Enhanced Layer-wise Deep Neural Networks (EL-DNN) and evaluate the performance and generalization ability of two architectures.	3839 LL radiographs (3063 in Data Set 1 + 776 in Data Set 2)	Canine thoracic radiographs from a single institution, two acquisition systems; labeled for multiple radiographic findings	Enhanced Layer-wise Deep Neural Networks (EL-DNN) based on ResNet-50 and DenseNet-121	Consensus interpretation by three board-certified veterinary radiologists.	ResNet-50 achieved AUC > 0.8 for most findings (e.g., alveolar pattern, cardiomegaly) except bronchial/interstitial patterns. ResNet-50 outperformed DenseNet-121 in generalization for alveolar pattern, interstitial pattern, megaoesophagus, and pneumothorax.	Potential to assist both general practitioners and specialists in daily clinical workflow, improving efficiency and diagnostic accuracy, especially in settings with limited access to board-certified radiologists.
Tahghighi et al. (2024) [39]	To develop a machine learning model for classifying the quality of canine and feline thoracic radiographs based on positioning, collimation, and exposure.	899 radiographs (889 dogs, 10 cats)	Dorsoventral and ventrodorsal thoracic radiographs	UNETR for segmentation; SVM, EfficientNet, Random Forest for classification of positioning, exposure, and collimation.	Binary quality labels (accepted/rejected) assigned by a single board-certified veterinary radiologist with >3 years of experience.	Model achieved an overall F1 score of 91.33 and AUC of 91.10. Positioning, collimation, and exposure were classified separately with high accuracy, enabling automated quality assessment.	Potential to reduce non-diagnostic images, improve radiographic quality, support clinical decision-making, and reduce costs by preventing repeat imaging.
Arsomngern et al. (2019) [40]	To develop a computer-aided diagnosis system (Pet-X) for detecting and classifying lung lesions in companion animals from thoracic X-ray images.	2862 thoracic X-ray image sets (ventrodorsal and lateral positions)	Thoracic X-ray images of dogs and cats with labels: Alveolar, Bronchial, Interstitial, or Normal	DenseNet-121-based CNN for detection and classification; SSD for lung cropping; Class Activation Mapping for localization.	Radiological reports by animal radiologists at the Faculty of Veterinary Medicine, Kasetsart University.	Achieved 79.6% accuracy in detecting abnormal lungs and 72.3% accuracy in classifying lesion types. Localized abnormalities with moderate success in lateral views.	Assists veterinarians in diagnosing lung lesions, especially in settings with limited radiology expertise. Can improve diagnostic accuracy and reduce misinterpretation.
Mekonnen et al. (2025) [41]	To develop an automated deep learning framework for estimating Vertebral Heart Size (VHS) and Cardiothoracic Ratio (CTR) from thoracic radiographs in dogs and cats.	343 radiographs (199 LM, 200 VD)	Lateral (LM) and ventrodorsal (VD) thoracic radiographs	Mask R-CNN for segmentation; YOLOv8 for region cropping; automated measurement of VHS (using T6 vertebra) and CTR.	Manual annotations by veterinary specialists (including radiologists, anesthesiologists, neurologists) from two clinical centers.	Achieved strong correlation with manual measurements (Pearson: 0.922 for VHS, 0.933 for CTR). First study to automate CTR estimation in veterinary radiology.	Provides objective, reproducible cardiac biomarkers to aid detection of cardiomegaly, especially in settings with limited radiology expertise. Enhances consistency and reduces inter-observer variability.
Dumortier et al. (2022) [9]	To develop a CNN-based approach for detecting radiographic pulmonary patterns (RPP) from lateral thoracic radiographs in cats.	500 radiographs (250 normal, 250 abnormal)	Lateral thoracic radiographs of cats, manually segmented intrathoracic region	ResNet50V2 with transfer learning from ImageNet and human CXRs; ensemble voting from 200 models; Grad-CAM for interpretation.	Medical Imaging Report (MIR) reviewed by at least one board-certified veterinary radiologist (ECVDI).	Achieved 82% accuracy, 85% F1-score, 88% sensitivity. Manual segmentation improved performance; ensemble voting and Grad-CAM enhanced reliability and interpretability.	Assists veterinarians in detecting lung abnormalities, especially in clinics without specialist radiologists. Provides visual explanations (heatmaps) to aid diagnosis and reduce misinterpretation.
Peyman Tahghighi et al. (2023) [42]	To classify appropriate vs. inappropriate collimation of cranial and caudal borders in canine/feline thoracic radiographs using machine learning.	900	Canine and feline ventrodorsal/dorsoventral thoracic radiographs from PACS (Ontario Veterinary College).	UNETR (CNN + Vision Transformer) for segmentation; MLP for classification; 5-fold cross-validation.	Assessment by a veterinary radiologist (>3 years experience) labeling each image as appropriately or inappropriately collimated at cranial and caudal borders.	Combined model achieved precision 91.21%, accuracy 83.17%, F1 score 87%. Model can assess inclusion of lung fields automatically.	Can be deployed clinically to improve radiograph quality, reduce repeat imaging, and assist veterinary staff in real-time collimation assessment.
S. Burti et al. (2020) [43]	To develop a CNN-based CAD method to detect cardiomegaly from canine thoracic radiographs.	1468 (1153 training + 315 test)	Right lateral canine thoracic radiographs from a veterinary teaching hospital (2014–2019).	Four CNN architectures tested: Inception V3, Inception-ResNet V2, VGG-19, ResNet-101; pre-trained on ImageNet.	Classification based on breed-specific Vertebral Heart Scale (VHS) reference intervals, measured by two experienced operators, as normal (No-VHS-Cardiomegaly) or enlarged (VHS-Cardiomegaly).	All models showed AUC > 0.9. Best model (ResNet-101) achieved AUC 0.973. CNNs can detect cardiomegaly with high accuracy using VHS as reference.	Can assist non-specialist veterinarians in detecting cardiomegaly, reduce interpretation errors, and improve diagnostic confidence, especially in emergency or general practice settings.
Jurgas et al. (2025) [44]	Develop an AI-based algorithm for automated segmentation of canine pulmonary masses in CT scans	217 cases (187 training/ validation, 30 test)	Canine thoracic CT scans with masses >2 cm from multiple institutions	nnUNet v2 framework with 3D Residual Encoder U-Net (ResEnc L)	Manual segmentation masks created by experts using open-source software 3D Slicer as ground truth.	Achieved mean Dice Similarity Coefficient (DSC) of 0.91 and Average Symmetric Surface Distance (ASSD) of 1.88 mm on test set. Model performed well on homogeneous, well-defined masses but struggled with intralesional mineralization and pleural effusion.	Provides a time-saving, reproducible, and scalable tool for segmenting lung masses in dogs, aiding in diagnosis, treatment planning, and longitudinal monitoring in clinical and teleradiology settings.
Norena et al. (2025) [45]	Evaluate a deep active learning model for segmenting canine thoracic radiographs.	50 radiographs; 22 participants	Canine thoracic radiographs (VD/DV)	UNET-Transformers (UNETR) with ViT encoder	Manual segmentation masks created by a board-certified veterinary radiologist with >3 years of experience, used as the reference standard for the anatomical structures (heart, spinous processes, abdomen).	Semiautomatic segmentation improved IoU and reduced Hausdorff distance vs. manual; improved repeatability (ICC 0.81 vs. 0.36); reduced time for experts.	VISTA tool enhances segmentation accuracy and efficiency for users of varying experience, reduces workload, and can be integrated into clinical workflows.
Kim et al. (2022) [46]	Determine the accuracy, sensitivity, and specificity of an AI-based software for diagnosing canine cardiogenic pulmonary edema (CPE) from thoracic radiographs	481 analyzable cases (out of 500 consecutive cases)	Canine thoracic radiographs (DICOM) acquired after-hours in an emergency setting	Convolutional Neural Networks (CNNs)—Deep Learning	Diagnosis by an American College of Veterinary Radiology (ACVR)-certified veterinary radiologist, who provided a binary classification (CPE+ or CPE-) blinded to patient data and AI results.	AI software achieved 92.3% accuracy, 91.3% sensitivity, and 92.4% specificity compared to a board-certified veterinary radiologist. High negative predictive value (99%) but lower positive predictive value (56%).	AI can serve as a reliable screening tool for CPE when a radiologist is unavailable, especially due to its high NPV. Can assist in urgent decision-making but positive findings should be confirmed by a specialist.
Celniak et al. (2023) [47]	Improve classification of veterinary thoracic radiographs via inter-species/inter-pathology self-supervised pre-training.	Pre-training: 511,876 images (human & canine); Fine-tuning: 12,416 vet images	Multi-source X-rays (human & canine), veterinary thoracic radiographs	Beta-VAE, Soft-Intro-VAE, SimCLR (self-supervised), CNN encoder	Annotations by three veterinary radiologists in consensus.	Self-supervised pre-training improved multi-label classification (mean ROC AUC: LL = 0.77, DV = 0.66). Enables knowledge transfer across species/pathologies.	Reduces need for large labeled vet datasets; improves diagnostic AI accuracy; supports One-Health cross-species applications.

**Table 6:** Studies related to AI to support multimodal-based diagnosis of respiratory diseases in dogs and cats.

Authors/Year	Goal	Sample	Data	Technique	Reference Exam	Contribution	Implications
Withington et al. (2021) [48]	To classify pressure sensor data from medical detection dogs’ sniffing behavior using ANNs	624 samples for development, 223 for final test	Univariate time series pressure sensor data (500 Hz) from sample pots	MLP, CNN, FCN, ResNet (tuned and untuned)	Known ground truth of sample positivity (amyl acetate concentration)	Not acoustic-based; uses pressure sensors. However, the paper mentions that stethoscopes were used in related work to extract acoustic features of sniffing behavior.	Demonstrates how AI can supplement or replace dogs’ operant responses (sitting), reducing cognitive load and training time. Could lead to automated, non-invasive diagnostic support systems in veterinary and medical detection settings.
Jarkoff (2023) [49]	Assess the accuracy of a smart collar for dogs in measuring resting heart and breathing rates	40 dogs	Motion sensor data from collar + ECG data + annotated video	K-fold cross-validation, custom AI algorithms	For Heart Rate (HR) and Pulse Detection: Portable ECG device (sampled at 120 Hz), with semi-automatic annotation of R-peaks and manual expert review. For Breathing Rate (BR): High-definition video recordings synchronized with sensor data, annotated by trained experts observing chest movements to count breaths per minute.	High accuracy in heart rate (SMAPE 0.38%) and breathing rate (SMAPE 1.42%) estimation; effective pulse detection (F1 score 98.04% at 50ms)	Enables continuous, non-invasive monitoring of canine vital signs at home; supports the detection of cardiac and respiratory issues.
Angelucci et al. (2025) [50]	Evaluate smartphone-based nearable methods (audio/video) for non-invasive respiratory rate monitoring in sleeping dogs	27	Audio and video recordings from sleeping dogs	Signal processing in MATLAB 2022b and 2023b; automated peak detection; AI-assisted ROI selection for video; peakfinder function	Manual breath counting by researchers as the reference standard: visual counting of inspiration peaks in synchronized 60-s audio/video recordings, performed by trained personnel without automation.	Video-based methods (especially lateral view) performed best (RMSE = 1.1, MAE = 0.7). Audio methods also accurate but more sensitive to noise. No significant difference between methods, but video more robust.	Enables low-cost, at-home, non-invasive respiratory monitoring using smartphones; useful for the detection of respiratory/cardiac issues; supports telemedicine and preventive care.
Foster et al. (2021) [51]	To reconstruct ECG and respiration signals from an IMU at various collar orientations during rest or sleep for dog welfare monitoring	1 dog (multiple positions)	IMU accelerometer signals, reference ECG, reference respiration belt data	Feedforward neural network for position classification; attention-based DNN for ECG reconstruction; CNN for respiration reconstruction	ECG: Custom wireless heart rate sensor system (WHRSS); Respiration: Vernier Go Direct respiration belt (strain gauge).	A multimodal pipeline was developed to first classify collar position, then apply position-specific models to reconstruct physiological signals. Achieved average HR accuracy 0.9131, RR accuracy 0.8932. Demonstrates feasibility of using a single IMU for multimodal physiological monitoring in sleeping dogs.	Enables non-invasive, long-term monitoring of heart and respiration rates in resting dogs using a wearable collar; supports welfare assessment and training outcome prediction for working dogs.
Chetboull et al. (2025) [52]	To establish a large reference database of resting HR and RR in apparently healthy dogs using a biometric collar	703 dogs	Heart rate (HR) and respiratory rate (RR) from wearable collar	AI-based algorithms for signal analysis (seismocardiography)	Portable ECG (for HR validation) and manual counting from thoracic video recordings (for RR validation), as per the device’s validation study	Provides integrative, long-term cardiorespiratory reference ranges; demonstrates feasibility of AI-driven detection of abnormal patterns (e.g., pre-clinical heart failure)	Enables non-invasive, continuous monitoring in home settings; supports the detection of cardiorespiratory diseases; offers breed- and age-specific reference values.
Angelucci et al. (2024) [53]	To validate a novel wearable inertial measurement unit (IMU) system for accurate respiratory rate (RR) monitoring in dogs under static conditions	12 dogs (14 acquisitions)	Inertial motion data (accelerometer, gyroscope) from 3 IMUs	Signal processing with adaptive filtering, PCA, spectral analysis (Welch’s method)	Vital signs monitor (clinical grade) during anesthesia.	Mean RMSE of 1.68 bpm compared to clinical monitor; demonstrated feasibility of wearable IMU-based RR monitoring in dogs	Enables non-invasive, continuous RR monitoring in home/sleep settings; supports detection of respiratory compromise in cardiac patients.

## Data Availability

No new data were created or analyzed in this study. Data sharing is not applicable to this article.

## Abbreviations

The following abbreviations are used in this manuscript:

AI	Artificial Intelligence
AUC	Area Under the Curve
BOAS	Brachycephalic Obstructive Airway Syndrome
CAD	Computer-Aided Diagnosis
CNN	Convolutional Neural Network
CPE	Cardiogenic Pulmonary Edema
CT	Computed Tomography
CTR	Cardiothoracic Ratio
DL	Deep Learning
DT	Decision Tree
ECG	Electrocardiogram
ETT	Exercise Tolerance Test
FFT	Fast Fourier Transform

GRU	Gated Recurrent Unit
HR	Heart Rate
IMU	Inertial Measurement Unit
KNN	k-Nearest Neighbors
LFCC	Linear Frequency Cepstral Coefficients
MAE	Mean Absolute Error
MFCC	Mel-Frequency Cepstral Coefficients
ML	Machine Learning
MLP	Multilayer Perceptron
MRI	Magnetic Resonance Imaging
NLP	Natural Language Processing
PCA	Principal Component Analysis
RF	Random Forest
RNN	Recurrent Neural Network
ROC	Receiver Operating Characteristic
ROI	Region of Interest
RR	Respiratory Rate
SVM	Support Vector Machine
STFT	Short-Time Fourier Transform
VHS	Vertebral Heart Size
VT	Vision Transformer
